# Economic evaluation of AbobotulinumtoxinA vs OnabotulinumtoxinA in real-life clinical management of cervical dystonia

**DOI:** 10.1186/s40734-020-0083-0

**Published:** 2020-02-11

**Authors:** V. P. Misra, N. Danchenko, P. Maisonobe, J. Lundkvist, M. Hunger

**Affiliations:** 1 Imperial College Healthcare NHS Trust, London, UK; 2 Ipsen, Boulogne–Billancourt, France; 3 Ipsen, Stockholm, Sweden; 4 ICON plc, Munich, Germany

**Keywords:** Cervical dystonia, Botulinum toxin, Observational study, Economic evaluation

## Abstract

**Background:**

Botulinum neurotoxins type A (BoNT-As) are commonly used treatments for cervical dystonia (CD). Clinical trials have demonstrated the benefits of them in these patients, but data from real-life clinical practice as well as comparative data on the cost and outcome of different BoNT-A formulations are limited. The aim of this study was to compare abobotulinumtoxinA (aboBoNT-A) and onabotulinumtoxinA (onaBoNT-A) on their clinical outcomes and drug costs in real-life clinical practice.

**Methods:**

This analysis included 356 adult patients with idiopathic CD treated with aboBoNT-A (*n* = 253) or onaBoNT-A (*n* = 103) from 38 centres across Europe and Australia (NCT00833196). The clinical outcome measures were treatment responses, changes in TWSTRS scores and changes in health utility scores from baseline to study visit 2 and 3. Health utility score was mapped from the TWSTRS total scale, using a previous publication. Costs included drug cost for France.

**Results:**

The aboBoNT-A treated group had 2.06 (95% CI: 1.15 to 3.69) times higher odds of achieving treatment response than the onaBoNT-A treated group. The adjusted mean change in TWSTRS total score from baseline to visit 3 were − 6.42 (95% CI: − 7.52 to − 5.33) for aboBoNT-A and − 3.94 (95% CI: − 5.68 to − 2.2) for onaBoNT-A, with a difference of − 2.48 (95% CI: − 4.57 to − 0.39). The corresponding difference in the adjusted mean change for health utility score was 0.008 (95% CI: 0.001 to 0.014). Mean treatment costs for aboBoNT-A and onaBoNT-A were 314.1 (95% CI: 299.1 to 329.0) and 346.6 (95% CI: 322.9 to 370.4) Euros, respectively.

**Conclusions:**

This comparative analysis indicated that treatment with aboBoNT-A may be less costly and lead to improved clinical outcomes when compared with onaBoNT-A, from a French healthcare system perspective. Additional comparative clinical data from larger patient cohorts, as well as more information about cost consequences of an improvement in clinical outcome would be of value to further confirm the findings.

## Introduction

Cervical dystonia (CD) or spasmodic torticollis is the most common type of focal dystonia with a prevalence ranging from 57 per million in Europe to as high as 280 per million in the USA [1–3]. It is characterized by sustained or intermittent involuntary contraction of the cervical muscles. This causes repetitive or tremulous movements of the head, neck and/or shoulder, and leads to painful and disabling abnormal postures [4]. The most frequent symptoms are neck twisting and lateral head rotation.

Majority of CD cases are idiopathic and there is currently no etiologic-based cure for CD. Nevertheless, symptomatic treatments are available for managing CD. A common first-line treatment for CD is injection of botulinum neurotoxin type A (BoNT-A) into the affected muscles [3, 5]. BoNT-A is a neurotoxin isolated and purified from the *Clostridium botulinum* type A bacteria. BoNT-A toxin causes muscle paralysis by inhibiting the peripheral cholinergic nerves from releasing acetycholine which triggers contraction of muscles. Several studies have shown that BoNT-A therapy increases free movement range, alleviates pain and improves quality of life for CD patients [6–9]. A considerable amount of studies have confirmed that BoNT-A is effective and well-tolerated in patients with CD [3]. Therefore, BoNT-A is now widely considered as an established, well characterised and effective treatment for CD.

Currently there are 3 commonly available formulations of BoNT-A: abobotulinumtoxinA (aboBoNT-A), onabotulinumtoxinA (onaBoNT-A) and incobotulinumtoxinA. Some comparative pharmacological data suggest that these formulations are not inter-changeable because they differ in their production process and potencies, [10, 11] and may not necessarily provide similar clinical outcome [12–15]. Comparative analyses between different BoNT-A formulations involve the issue of determining appropriate doses to compare. Furthermore, such studies are primarily based on randomised clinical trial (RCT) findings which involve specifically selected patient subjects and may not be applicable to real-world CD patient population.

With the expanding clinical use of different formulations of BoNT-A, there is a need of data for better differentiation of the available formulations, especially in terms of their treatment outcome and cost-effectiveness in real-world clinical setting. Such information are crucial for guiding healthcare decision makers and understanding costs and health gains of alternative interventions.

The INTEREST IN CD1 (CD1) study was an international, multicentre, noninterventional, prospective, longitudinal study (NCT00833196) with primary objective to assess responder rate following one BoNT-A injection cycle [16]. The objective of the present post-hoc analysis of the CD1 observational study is to compare abobotulinumtoxinA (aboBoNT-A) and onabotulinumtoxinA (onaBoNT-A) on clinical outcomes and drug cost in the treatment of CD.

## Subjects and methods

### Study design

The observational, prospective, longitudinal study enrolled 404 subjects from 38 centres across Europe and Australia (NCT00833196). Eligible subjects were: ≥18 years old, suffering from idiopathic CD with a Toronto Western Spasmodic Torticollis Rating Scale (TWSTRS) severity score ≥ 15, and a ≥ 12 week interval between the last injection (BoNT-A or BoNT-B) and study inclusion.

The decision to prescribe a BoNT-A preparation was to be taken prior to, and independently from, the decision to enrol the subject. The prescribing of a BoNT-A preparation was to be made in accordance with routine clinical practice at the centres concerned. Investigators were free to choose the targeted muscles, BoNT-A preparation, injected dose, number of points and volume per point. In order to avoid bias in the recruitment of subjects, physicians were not allowed to choose their subjects but were asked to include consecutive subjects during BoNT-A consultations.

Subjects were assessed during their usual centre visits, which were to be in line with the current practice at the centre. Study visits needed to include an inclusion visit (visit 1), a follow-up visit (visit 2) 3 to 6 weeks after injection, and an end of study visit (visit 3) 12 to 16 weeks after injection. If the follow-up visit (3 to 6 weeks after BoNT-A treatment) was not part of the centres usual practice, then it was not performed. The subject was to contact the investigator at the time when he/she felt that there was a clinically relevant waning of treatment effect justifying a re-injection. Further details on study design can be found elsewhere [16].

### Study population

The study population for this analysis consists of 367 patients with data for each of the four criteria required in the initial definition of response. These criteria include having data for TWSTRS severity score at baseline and visit 2 or visit 3, assessment of whether there were any related adverse events (AEs) at visit 2 or visit 3, data for the subject’s Clinical Global Improvement (CGI) at visit 2 and documentation as to whether there was a waning of treatment effect.

The specific study population for the comparative analyses described here comprised of adult patients treated with aboBoNT-A (*n* = 253) or onaBoNT-A (*n* = 103) from the original efficacy population, excluding 11 patients treated with incobotulinumtoxinA.

### Outcomes

The primary efficacy outcome of the study was treatment response. Two definitions of responders were used in this post-hoc analysis. The initial definition of responder was: ≥25% improvement in TWSTRS severity scores at visit 2, at least a 12 week interval between injection and subject report of treatment waning, no report of related severed AEs at visit 2 or visit 3, and a CGI score equal to either + 2 or + 3 at visit 2. An alternative definition of responder was: ≥25% improvement in TWSTRS severity scores at visit 3, and no report of a severe AE related to BoNT-A injection at visit 3. This alternative response variable was included since there is no agreed definition of treatment response and results may vary depending on definition used.

Secondary efficacy outcome included change in TWSTRS scores. The TWSTRS is an assessment of CD that includes a total CD rating score and three domain scores (Torticollis Severity, Disability and Pain). For the TWSTRS total and subscale scores, a lower value represents a better outcome. Change in health utility score from baseline to visit 3 was considered as an additional exploratory endpoint in this post-hoc analysis. Health utility was expressed based on the EQ-5D. The EQ-5D was not directly administered to patients. Instead, it was calculated from a simple mapping algorithm described in a previously published paper (see Jen et al. 2014), which estimated the linear association between the TWSTRS total score and EQ-5D health utilities [17]. CD patients in the Jen study were administered both the TWSTRS, and the Short Form-36 (SF-36) Health Survey. In a first step, patients’ responses to the SF-36 were mapped to the EQ-5D, using an adapted version of the algorithm of Rowen et al. [18] In a second step, the mapped EQ-5D utilities were regressed to TWSTRS total scores using a generalized linear model. Data from Fig. 2 of Jen et al. were digitized using the software DigitizeIt (DigitizeIt, Braunschweig, Germany), and the regression line was estimated to be characterized by the following equation: *y*_*EQ* − 5*D*_ = 0.6811 − 0.003136 × *x*_*TWSTRS*._

Cost of treatment with BoNT-A was calculated by valuing patients’ health care resource utilization with corresponding unit costs. A French health care provider perspective was applied focusing on drug costs only. Drug costs were based on retail prices without commercial offer [19], and were calculated by multiplying the total units injected at the inclusion visit with the average drug cost per unit. The following costs per unit of aboBoNT-A and unit of onaBoNT-A were used: 0.5182 € per unit of aboBoNT-A (259.16 € per 500 U) and 2.0581 € per unit of onaBoNT-A (411.62 € per 200 U).

The cost per responder (CPR) concept describes the relative value of two drugs by comparing the cost per patient relative to achieving a defined response level. Both treatment response definitions are applied in the CPR analysis. To calculate CPR, mean total treatment costs are divided by the response rates in each of the two treatment groups.

### Statistical analyses

To minimize the risk of confounding, all unadjusted comparisons of cost and outcomes were complemented by adjusted comparisons obtained from multiple regression models. Logistic regression was used to compare responder rates between treatment groups. Adjusted effects of treatment were summarized by the odds ratio together with 95% confidence intervals. Linear regression models were used to analyse cost and change in TWSTRS total/subscale and health utility scores. Least-square means of cost and TWSTRS/ health utility change scores were calculated by treatment group. Least-square means correspond to marginal means over a balanced population – holding the other covariates in the model constant at their mean. The following list of variables was considered for potential inclusion in the regression models: age (continuous), sex, body weight (continuous), previous surgery, prior treatment with BoNT-A, presence of tremor, predominant component (rotation vs. not rotation), and use of electromyography (EMG) at baseline

A three-step approach was followed for model selection to avoid overfitting and collinearity: (1) Any potential covariate must have had a *p*-value < 0.2 in a univariate analysis, i.e. a regression model including that variable only. (2) In case of highly significant correlation of *p* < 0.001 between a pair of variables, only one of the two concerned variables was selected based on clinical rationale. (3) A multivariate backward regression analysis starting with all variables after the two previous steps was performed removing all variables with *p* > 0.05. Sex and prior treatment with BoNT-A was forced to be included in all adjusted models to account for potential differences in disease duration. For reasons of consistency, the models on change from TWSTRS total and TWSTRS subscale scores used the same set of covariates, i.e. variable selection was only performed for the models on change from baseline in TWSTRS total score.

For the unadjusted CPR, mean treatment cost were divided by the response rate in each of the two treatment groups. For the adjusted CPR, adjusted mean total costs were divided by adjusted response rates in each of the two treatment groups. Adjusted mean total cost and adjusted response rates were obtained from linear regression models, controlling for sex, prior treatment with BoNT-A and EMG at baseline.

## Results

### Baseline characteristics

Table 1 summarises the patient demographics and disease history by treatment group. The largest age group for patients receiving aboBoNT-A was 41–50 years (29.2%), while the largest age group of patients receiving onaBoNT-A was 51–60 years (35%). The treatment groups were comparable in terms of sex and body weight. The aboBoNT-A group had a lower percentage of patients receiving prior BoNT-A treatment (80.6% vs. 93.2%), lower percentage of tremor presence (43.9% vs. 60.2%), and greater percentage of rotation being the predominant component (80.6% vs. 63.1%). Patients treated with aboBoNT-A had slightly higher TWSTRS scores at baseline than patients treated with onaBoNT-A. The average injected dose of BoNT-A for the aboBoNT-A group was 598.8 units and for the onaBoNT-A group was 172.9 units.

**Table 1:** Subject demographics and baseline characteristics

Patient Characteristics	AboBoNT-A (*N* = 253)	OnaBoNT-A (*N* = 103)	All Patients (*N* = 356)
Age in Years			
18 to 30	13 (5.1%)	4 (3.9%)	17 (4.8%)
31 to 40	37 (14.6%)	9 (8.7%)	46 (12.9%)
41 to 50	74 (29.2%)	18 (17.5%)	92 (25.8%)
51 to 60	58 (22.9%)	36 (35%)	94 (26.4%)
61 to 70	45 (17.8%)	26 (25.2%)	71 (19.9%)
> 70	26 (10.3%)	10 (9.7%)	36 (10.1%)
Body Weight (kg), mean (SD)	72.7 (14.9)	73.7 (15.3)	73.0 (15.0)
Sex (n,%)			
Female	162 (64%)	69 (67%)	231 (64.9%)
Male	91 (36%)	34 (33%)	125 (35.1%)
Previous Surgery (n,%)			
No	248 (98%)	100 (97.1%)	348 (97.8%)
Yes	5 (2%)	3 (2.9%)	8 (2.2%)
Prior Treatment with BoNT-A (n,%)			
No	49 (19.4%)	7 (6.8%)	56 (15.7%)
Yes	204 (80.6%)	96 (93.2%)	300 (84.3%)
Tremor (n,%)			
Absent	142 (56.1%)	41 (39.8%)	183 (51.4%)
Present	111 (43.9%)	62 (60.2%)	173 (48.6%)
Predominant Component (n,%)			
Not Rotation	49 (19.4%)	38 (36.9%)	87 (24.4%)
Rotation	204 (80.6%)	65 (63.1%)	269 (75.6%)
EMG at Baseline (n,%)			
No	151 (59.7%)	40 (38.8%)	191 (53.7%)
Yes	102 (40.3%)	63 (61.2%)	165 (46.3%)
TWSTRS Scores at Baseline			
Total Score, mean (SD)	37.8 (9.4)	33.9 (11.1)	37.1 (10.2)
Severity Subscale Score, mean (SD)	20.1 (3.5)	18.9 (3.4)	19.8 (3.5)
Disability Subscale Score, mean (SD)	11.1 (5.3)	9.2 (6.0)	10.7 (5.6)
Pain Subscale Score, mean (SD)	6.6 (4.7)	5.8 (4.9)	6.6 (4.8)
EQ-5D Utility Score at Baseline, mean (SD)	0.56 (0.03)	0.57 (0.03)	0.57 (0.03)
Total Injected Units of BoNT-A, mean (SD)	598.8 (231.9)	172.9 (66.5)	–

*SD* Standard deviation; *EMG* electromyography; *BoNT-A* Botulinum Neurotoxin type A; *TWSTRS* Toronto Western Spasmodic Torticollis Rating Scale; *EQ-5D* EuroQol 5 Dimensions Questionnaire

### Comparison of clinical outcomes

#### Treatment response

Patients treated with aboBoNT-A had a higher response rate than patients treated with onaBoNT-A, regardless of whether the initial definition of response (32.0% vs. 22.3%) or the supportive definition of response (33.9% vs. 19.4%) was used. The estimated association between treatment and response after adjusting for covariates is shown in Table 2. Based on the initial definition of response, aboBoNT-A increased the odds of response by 67% compared to onaBoNT-A, though the effect was not statistically significant at 0.05 level (OR 1.67; 95% CI: 0.97 to 2.87; *p* = 0.0629). In the analysis of the supportive definition of treatment response, a statistically significant two-fold higher odds of treatment response was observed with aboBoNT-A compared to onaBoNT-A (OR 2.06; 95% CI: 1.15 to 3.69; *p* = 0.0148).

**Table 2:** Association between treatment groups (aboBoNT-A versus onaBoNT-A)^a^ and treatment responses

Treatment Responses	Unadjusted OR (95%CI)	Adjusted OR (95%CI)
Primary Endpoint Analysis ^b^	1.64 (0.96, 2.79), *p* = 0.0700	1.67 (0.97, 2.87)^d^, *p* = 0.0629
Alternative Endpoint Analysis^c^	2.12 (1.22, 3.7), *p* = 0.0076	2.06 (1.15, 3.69) ^e^, *p* = 0.0148

^a^Reference group is onaBoNT-A

^b^Response is defined as: ≥25% improvement in TWSTRS severity scores at visit 2, at least a 12 week interval between injection and subject report of treatment waning, no report of related severed adverse events a visit 2 or visit 3, and a Clinical Global Improvement (CGI) score equal to either + 2 or + 3 at visit 2

^c^Response is defined as: ≥25% improvement in TWSTRS severity scores at visit 3, and no report of a severe adverse event related to BoNT-A injection at visit 3

^d^Odds ratio adjusted for sex and prior treatment with BoNT-A

^e^Odds ratio adjusted for age, sex and prior treatment with BoNT-A

*OR* odds ratio; *95%CI* 95% confidence interval; *BoNT-A* Botulinum Neurotoxin type A

#### Change in TWSTRS scores

The analysis of change in TWSTRS total score from baseline to visit 2 and visit 3 showed that aboBoNT-A had a greater improvement as compared to onaBoNT-A (Table 3). The difference in change scores was − 4.80 (95% CI: − 7.10, − 2.50) at visit 2 and − 3.62 (95% CI: - 5.68, − 1.56) at visit 3. The differences (− 3.16 at visit 2, 95% CI: − 5.49 to − 0.84; − 2.48 at visit 3, 95% CI: − 4.57 to − 0.39) remained statistically significant after adjusting for baseline TWSTRS total score and baseline characteristics including sex, age and prior treatment with BoNT-A. For change from baseline to visit 2, change scores were also adjusted for presence of tremor and EMG at baseline. Change in TWSTRS subscale scores (i.e. severity, disability and pain) from baseline to visit 2 and visit 3 also showed similar trend as TWSTRS total score, with a better improvement or greater decrement in all three subscale scores in the group treated with aboBoNT-A.

**Table 3:** Change in TWSTRS scores and health utility score from baseline to visit 2 and visit 3

	Unadjusted Model			Adjusted Model^a^		
	AboBoNT-A LS Mean (95% CI)	OnaBoNT-A LS Mean (95% CI)	Difference^b^ (95% CI)	AboBoNT-A LS Mean (95% CI)	OnaBoNT-A LS Mean (95% CI)	Difference^b^ (95% CI)
Change from baseline to visit 2						
TWSTRS Scores						
Total	−15.80 (− 17.04, − 14.57)	−11.00 (− 12.94, − 9.07)	−4.80 (− 7.10, − 2.50)	−15.33 (− 16.53, − 14.13)	−12.17 (− 14.09, − 10.24)	−3.16 (− 5.49, − 0.84)
Severity Subscale	−8.49 (− 9.08, − 7.90)	−6.75 (− 7.67, − 5.82)	−1.74 (− 2.84, − 0.64)	−8.30 (− 8.88, − 7.71)	−7.21 (− 8.15, − 6.27)	−1.08 (− 2.22, 0.05)
Disability Subscale	−4.56 (− 5.12, − 3.99)	−2.77 (− 3.65, − 1.88)	−1.79 (− 2.84, − 0.74)	−4.30 (− 4.81, − 3.79)	−3.39 (− 4.21, − 2.57)	− 0.91 (− 1.90, 0.08)
Pain Subscale	−2.76 (− 3.20, − 2.32)	−1.49 (− 2.18, − 0.80)	−1.27 (− 2.09, − 0.45)	− 2.67 (− 3.04, − 2.30)	− 1.71 (− 2.30, − 1.12)	−0.96 (− 1.68, − 0.25)
Health Utility Score	0.050 (0.046, 0.053)	0.035 (0.028, 0.041)	0.015 (0.008, 0.022)	0.048 (0.044, 0.052)	0.038 (0.032, 0.044)	0.010 (0.003, 0.017)
Change from baseline to visit 3						
TWSTRS Scores						
Total	−6.75 (− 7.87, −5.64)	− 3.13 (− 4.87, − 1.40)	−3.62 (− 5.68, − 1.56)	−6.42 (− 7.52, − 5.33)	− 3.94 (− 5.68, − 2.20)	−2.48 (− 4.57, − 0.39)
Severity Subscale	−3.70 (− 4.21, − 3.19)	−2.02 (− 2.81, − 1.22)	−1.68 (− 2.63, − 0.74)	−3.55 (− 4.06, − 3.05)	−2.38 (− 3.18, − 1.58)	−1.18 (− 2.14, − 0.22)
Disability Subscale	− 1.93 (− 2.43, − 1.42)	−0.63 (− 1.42, 0.16)	−1.30 (− 2.23, − 0.36)	−1.78 (− 2.27, − 1.29)	−1.00 (− 1.78, − 0.22)	−0.78 (− 1.71, 0.16)
Pain Subscale	−1.12 (− 1.59, − 0.66)	−0.48 (− 1.20, 0.24)	−0.64 (− 1.50, 0.21)	−1.04 (− 1.45, − 0.62)	−0.70 (− 1.36, − 0.04)	−0.34 (− 1.13, 0.45)
Health Utility Score	0.021 (0.018, 0.025)	0.010 (0.004, 0.015)	0.011 (0.005, 0.018)	0.020 (0.017, 0.024)	0.012 (0.007, 0.018)	0.008 (0.001, 0.014)

^a^For change from baseline to visit 2, the model is adjusted for baseline score, sex, prior treatment with BoNT-A, age, presence of tremor and use of EMG; for change from baseline to visit 3, the model is adjusted for baseline score, sex, prior treatment with BoNT-A and age

^b^Difference was calculated as follow: aboBoNT-A LS mean minus onaBoNT-A LS mean

*TWSTRS* Toronto Western Spasmodic Torticollis Rating Scale; *LS* Least Square; *CI* Confidence Interval; *SD* Standard deviation; *EMG* electromyography; *BoNT-A* Botulinum Neurotoxin type A

#### Change in utility score

The results from the regression analyses on change in utility score from baseline to visit 2 and visit 3 are shown in Table 3. The unadjusted model showed that aboBoNT-A had a statistically significant greater improvement in utilities score from baseline to visit 3 compared to onaBoNT-A (0.011 [95% CI 0.005, 0.018]). After adjusting for baseline utility score and baseline characteristics, the difference was 0.008 with a 95% CI of 0.001 to 0.014.

### Comparison of treatment cost

#### Drug cost

Table 4 provides a descriptive comparison of treatment cost by treatment group. Patients treated with aboBoNT-A had about 46 € lower treatment cost than patients treated with onaBoNT-A (310.31 € vs. 355.87 €). After adjusting for sex, prior treatment with BoNT-A and electromyography at baseline, this difference in cost was 32.57 € (95% CI: 4.09, 61.04).

**Table 4:** Cost^a^ of treatment by treatment group

	AboBoNT-A LS Mean (95% CI)	OnaBoNT-A LS Mean (95% CI)	Difference^b^ (95% CI)
Unadjusted Model	310.31 (294.83, 325.78)	355.87 (331.61, 380.13)	−45.57 (−74.35, − 16.79)
Adjusted Model^c^	314.07 (299.1, 329.03)	346.63 (322.85, 370.42)	−32.57 (− 61.04, − 4.09)

^a^Cost was calculated based on retail drug cost in euros

^b^Difference was calculated as: mean of aboBoNT-A minus mean of onaBoNT-A

^c^Model adjusted for sex, prior treatment with botulinum neurotoxin type A and electromyography (EMG) at baseline

*aboBoNT-A* abobotulinumtoxinA; *onaBoNT-A* onabotulinumtoxinA; *LS* Least-Square; *CI* Confidence Interval

#### Cost-per-responder

Table 5 summarises cost-per-responder analysis by treatment group, unadjusted and adjusted for age, sex, prior treatment with BoNT-A and use of EMG at baseline. The results showed that the adjusted mean total cost-per-responder was lower for patients receiving aboBoNT-A compared to patients receiving onaBoNT-A (418 € vs. 496 €).

**Table 5:** Cost-per-responder analysis

Primary Response Analysis^a^			
	AboBoNT-A (*N* = 253)	OnaBoNT-A (*N* = 103)	All Patients (*N* = 356)
Unadjusted			
Mean Total Cost (€)	310.31	355.87	323.49
Mean Response Rate	0.32	0.22	0.29
Mean Cost-Per-Responder	969.23	1593.69	1107.33
Adjusted^b^			
Mean Total Cost (€)	314.18	346.35	
Mean Response Rate	0.32	0.23	
Mean Cost-Per-Responder	986.35	1523.77	
Alternative Response Analysis^c^			
	AboBoNT-A (*N* = 251)	OnaBoNT-A (*N* = 103)	All Patients (*N* = 354)
Unadjusted			
Mean Total Cost (€)	310.30	355.87	323.56
Mean Response Rate	0.34	0.19	0.30
Mean Cost-Per-Responder	916.30	1832.75	1090.86
Adjusted^b^			
Mean Total Cost (€)	314.30	346.12	
Mean Response Rate	0.34	0.20	
Mean Cost-Per-Responder	931.45	1755.83	

^a^Response is defined as: ≥25% improvement in TWSTRS severity scores at visit 2, at least a 12 week interval between injection and subject report of treatment waning, no report of related severed adverse events a visit 2 or visit 3, and a Clinical Global Improvement (CGI) score equal to either + 2 or + 3 at visit 2

^b^Adjusted for age, sex, prior treatment with BoNT-A, and use of EMG at baseline

*aboBoNT-A* abobotulinumtoxinA; *onaBoNT-A* onabotulinumtoxinA

^c^Response is defined as: ≥25% improvement in TWSTRS severity scores at visit 3, and no report of a severe adverse event related to BoNT-A injection at visit 3

## Discussion

In this non-interventional study, we observed that patients treated with aboBoNT-A have greater clinical outcome improvements from baseline to study visit 3 (i.e. 12 to 16 weeks after injection) than patients treated with onaBoNT-A. This observation is supported by a higher chance of achieving treatment response, and a larger improvement in both TWSTRS scores and health utility score. In addition, aboBoNT-A treatment group has lower average treatment drug cost and lower cost-per-responder.

This study uses multidimensional definitions of treatment response, taking into account that the assessment of effectiveness in the real-world setting cannot be limited to TWSTRS or Tsui scores only. The first definition was developed by an expert group of neurologists in 2008 and combines different aspects of efficacy, tolerability and assessment of global improvement [16]. The second definition is less stringent; it does not include the assessment of global improvement and takes into account that not all patients routinely have a follow-up visit three to six weeks after treatment administration. Regardless of which definition was applied, the aboBoNT-A treated group has better response rates than the onaBoNT-A treated group, albeit a statistical significant difference was observed only with definition 2.

This is a large-scale international study of CD patients and treatment of BoNT-A in a real-life clinical setting. Whereas RCTs have high internal validity due to randomization, they may often lack external validity because of highly selected patients which may not be representative of real-world patient populations. Also, the administration of BoNT-A in routine care may not reflect the standardised methods applied in clinical trials, because injection schemes are individually determined by the treating physician. This observational study adds to the existing body of evidence obtained from RCTs on the efficacy of different BoNT-A formulations by assessing comparative effectiveness between aboBoNT-A and onaBoNT-A in a real-life setting.

Patients with cervical dystonia often have concomitant medications such as muscle relaxants and benzodiazepines. A previous report that used the same data and study population as our current study showed that absence of concomitant medication at baseline may be associated with increased likelihood of treatment response, though the association was statistically non-significant [16]. If the proportion of patients with concomitant medications differs substantially between the treatment groups, then this could affect the comparison of outcomes. However, further investigation of the data reveals that the proportion of subjects without baseline concomitant medication between the two treatment groups were comparable (aboBoNT-A 47.0% versus onaBoNT-A 48.5%). The same previous report also observed that aboBoNT-A treated group and onaBoNT-A treated group had no statistical significant difference in adverse event frequency [16]. In fact, the percentage of patients without severe adverse events was relatively similar between the two treatment groups (aboBoNT-A 98.0% versus onaBoNT-A 97.1%). In the current study, adverse events was included as a component of treatment response definition, and some aspects of tolerability may also partly be captured by the utility score. These suggest that the observed better efficacy profile in aboBoNT-A group is unlikely to be coupled with a worse or inferior tolerability profile than onaBoNT-A.

The efficacy of BoNT-A in the treatment of CD has been investigated by several randomised clinical trials. A Cochrane systematic literature review analysed data from one trial involving onaBoNT-A formulation, [3, 20] five trials involving aboBoNT-A formulation and one trial involving incoBoNT-A [6, 8, 9, 21–23]. It found that all three formulations were efficacious against placebo (mostly based on TWSTRS scores), but that the efficacy against placebo of the three formulations did not differ significantly. In this observational study, we observed a statistically significant difference with regards to the change in TWSTRS scores and health utility score between aboBoNT-A and onaBoNT-A. In our study, a direct comparison between aboBoNT-A treated group and onaBoNT-A treated group was performed, whereas in the systematic review, comparison was made between the efficacies of each formulations found in separate studies, with the efficacy measured relative to placebo.

A few clinical trials have also been conducted head-to-head comparisons between aboBoNT-A and onaBoNT-A using different dose conversation units [12–15]. Two randomised crossover trials found significant difference between the two treatments [13, 14]. Ranoux et al. reported a significant better improvement in the aboBoNT-A group, with improvement defined as difference in TWSTRS pain score after 1 month. Rystedt et al. observed slightly better TWSTRS total scores at week 4 (non-significant) and week 12 (significant) in favour of aboBoNT-A, which is similar to our findings. Two other randomised trials, [12, 15] with one having a cross-over design and the other having a parallel group design, found similar efficacy between the two treatments, which is different to our findings. Notably, in those randomised trials, aboBoNT-A and onaBoNT-A were applied at a pre-specified dose conversion ratio intended to find a dosing equivalence between the different formulations. By contrast, in the current non-interventional study, physicians were free to choose the injected dose number of points and volume per point, which reflects real-life clinical practice on how BoNT-A treatment is given to CD patients. Current guidance recommends to consider a conversion of 1 IU onaBoNT-A to 3 IU aboBoNT-A [5]. The dose ratio based on the median dose in our study was 3.1, thus there is no evidence that patients in the aboBoNT-A group received higher doses than patients in the onaBoNT-A group.

Choosing a cost-effective treatment is also important to ensure efficient use of the medication. A retrospective cohort study conducted within a single U.S. private neurological practice reported that switching from onaBoNT-A to aboBoNT-A in the treatment of CD resulted in a mean reduction of 37% in both total reimbursement cost (procedure plus BoNT-A) and reimbursed BoNT-A cost alone [24]. This observation is in line with the findings of our larger multi-centre observational study, in which we observed that aboBoNT-A treatment incurred lower treatment drug cost than onaBoNT-A treatment. In addition to lower drug cost, aboBoNT-A treatment also leads to lower mean cost-per-responder as observed in our study. This finding is also consistent with a previous report in which a lower cost-effectiveness ratio for aboBoNT-A was observed as compared with onaBoNT-A ($36,678 per quality-adjusted life years (QALYs) versus $49,337 per QALYS) [25]. Overall, these strengthen the notion that aboBoNT-A is a more cost-effective treatment alternative than onaBoNT-A.

This study has several limitations. Our analyses only considered drug costs and did not take into account other cost components such as administration costs, electromyography costs, consultation costs and among others. This is likely lead to a conservative estimate of costs, since improved clinical outcome may potentially also lead to cost savings. Further investigations could consider additional treatment costs as part of the comparison. In addition, the costs reflect drug prices in France and this may imply that our findings on the treatment cost may not be generalizable to other geographic settings.

Another limitation is the simplicity of the mapping algorithm used to obtain utility scores from TWSTRS scores. A linear regression line was mapped based on data digitized from a single plot of observed utility scores against TWSTRS total scores only. Typical mapping algorithms are more complex, taking into account subscales scores and sometimes even demographic or clinical variables. The mapping algorithm used in our study has not been assessed for model performance and predictive power, therefore it is unclear how precise this simple method is in prediction utility values from TWSTRS scores.

Lastly, our observational study is based on a non-randomized setting which means that a causal relationship between the treatment and outcome cannot be drawn. Following good research practices on real-world evidence and comparative effectiveness research for decision making [26, 27], we fitted multiple regression models to adjust for a number of potential confounders. However, since only a few covariates were observed, the possibility of residual confounding cannot be ruled out, and relevant baseline characteristics and prognostic variables may not be balanced between treatment groups. This potential imbalance may have influenced the estimated differences in treatment outcomes and drug costs between the two groups. The two treatment groups differed with respect to baseline TWSTRS scores, suggesting that aboBoNT-A was prescribed more frequently to patients with more severe disease than onaBoNT-A. Our comparative analyses of TWSTRS and utility score adjusted for the difference in baseline scores. However, there is an ongoing debate on whether adjustments for baseline scores should be made in a non-randomized setting [28]. As a sensitivity analyses, we also fitted regression models that did not adjust for TWSTRS baseline values and found that results did not alter overall conclusions. The sample size in the aboBoNT-A treated group was more than twice as large as the sample size in the onaBoNT-A group, however, differences in group size do not systematically bias comparative analyses. Also, with more than 100 patients, the onaBoNT-A group still had a reasonable sample size to allow for meaningful and robust analyses.

## Conclusions

This study conducted comparative effectiveness analyses between two different formulations of botulinum neurotoxin type A for the treatment of cervical dystonia. The analyses indicated that abobotulinumtoxinA and onabotulinumtoxinA differ in their cost-effectiveness, with abobotulinumtoxinA incurring a lower treatment drug cost and greater clinical outcome improvement based on the change in TWSTRS scores from baseline. The findings from this non-interventional study reflect the cost-effectiveness of the two formulations in real-life clinical management of cervical dystonia. This could have potential implications in identifying cost-effective care for both patients and related healthcare systems.

## Data Availability

The data that support the findings of this study are available from Ipsen, Boulogne-Billancourt, France, but restrictions apply to the availability of these data, which were used under license for the current study, and so are not publicly available.

## Abbreviations

aboBoNT-A: AbobotulinumtoxinA
AE: Adverse event
BoNT-A: Botulinum neurotoxins type A
CD: Cervical dystonia
CGI: Clinical Global Improvement
CI: Confidence interval
CPR: Cost per responder
EMG: Electromyography
onaBoNT-A: OnabotulinumtoxinA
OR: Odds ratio
RCT: Randomised clinical trial
SF-36: Short Form-36 Health Survey
TWSTRS: Toronto Western Spasmodic Torticollis Rating Scale
