# Correction to: Economic evaluation of AbobotulinumtoxinA vs OnabotulinumtoxinA in real-life clinical management of cervical dystonia

**DOI:** 10.1186/s40734-020-00088-5

**Published:** 2020-07-29

**Authors:** V. P. Misra, N. Danchenko, P. Maisonobe, J. Lundkvist, M. Hunger

**Affiliations:** 1 Imperial College Healthcare NHS Trust, London, UK; 2 Ipsen, Boulogne–Billancourt, France; 3 Ipsen, Stockholm, Sweden; 4 ICON plc, Munich, Germany

**Correction to: J Clin Mov Disord 7, 2 (2020)**

**https://doi.org/10.1186/s40734-020-0083-0**

Following publication of the original article [1], an error was identified in the *Cost-per-responder* sub-section.

The updated conclusion is given below and the changes have been highlighted in **bold typeface**.

The results showed that the adjusted mean total cost-per-responder was lower for patients receiving aboBoNT-A compared to patients receiving onaBoNT-A (**€986 vs. €1524), based on the primary treatment response definition**.
